# Strategies for the Identification of Bioactive Neuropeptides in Vertebrates

**DOI:** 10.3389/fnins.2019.00948

**Published:** 2019-09-18

**Authors:** Auriane Corbière, Hubert Vaudry, Philippe Chan, Marie-Laure Walet-Balieu, Thierry Lecroq, Arnaud Lefebvre, Charles Pineau, David Vaudry

**Affiliations:** ^1^Normandie Univ, UNIROUEN, Inserm, Laboratory of Neuronal and Neuroendocrine Communication and Differentiation, Neuropeptides, Neuronal Death and Cell Plasticity Team, Rouen, France; ^2^Normandie Univ, UNIROUEN, Regional Cell Imaging Platform of Normandy (PRIMACEN), Rouen, France; ^3^Normandie Univ, UNIROUEN, Rouen Proteomic Platform (PISSARO), Institute for Research and Innovation in Biomedicine (IRIB), Rouen, France; ^4^Normandie Univ, UNIROUEN, LITIS EA 4108, Information Processing in Biology & Health, Rouen, France; ^5^Protim, Univ Rennes, Rennes Cedex, France

**Keywords:** neuropeptide, identification, peptidomic approach, de novo, bioactiity, review

## Abstract

Neuropeptides exert essential functions in animal physiology by controlling *e.g.*, reproduction, development, growth, energy homeostasis, cardiovascular activity and stress response. Thus, identification of neuropeptides has been a very active field of research over the last decades. This review article presents the various methods used to discover novel bioactive peptides in vertebrates. Initially identified on the basis of their biological activity, some neuropeptides have also been discovered for their ability to bind/activate a specific receptor or based on their biochemical characteristics such as C-terminal amidation which concerns half of the known neuropeptides. More recently, sequencing of the genome of many representative species has facilitated peptidomic approaches using mass spectrometry and *in silico* screening of genomic libraries. Through these different approaches, more than a hundred of bioactive neuropeptides have already been identified in vertebrates. Nevertheless, researchers continue to find new neuropeptides or to identify novel functions of neuropeptides that had not been detected previously, as it was recently the case for nociceptin.

## Characteristics of Neuropeptides

**FIGURE 1:**
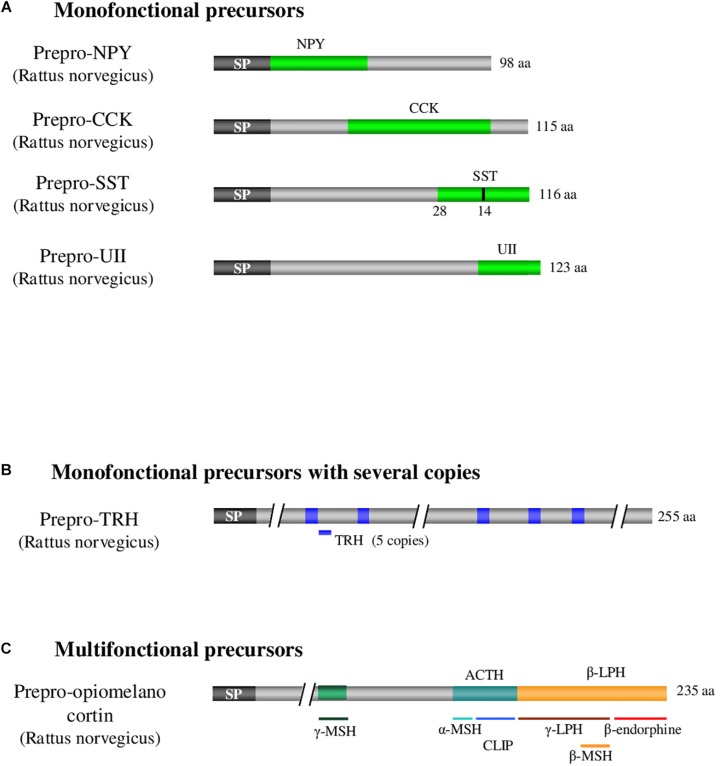
Examples illustrating the 3 categories of neuropeptide precursors. **(A)** Illustration of monofonctional precursors with single copy of the peptide of interest as it is the case for CCK, UII, NPY, and SST. **(B)** Illustration of monofonctional precursors with several copies of the peptide of interest as it is the case for TRH. **(C)** Illustration of multifonctional precursors which express several different bioactive peptides as it is the case with POMC. ACTH: adrenocorticotropic hormone. CCK: cholecystokinin. POMC: pro-opiomelanocortin. CLIP: corticotropin-like intermediate lobe peptide. LPH: lipotropic hormone. MSH: melanocyte-stimulating hormone. NPY: neuropeptide Y. SP: signal peptide. SST: somatostatin. TRH: thyrotropin-releasing hormone. UII: urotensin II. aa: aminoacid. Adapted from Neveu (2012).

More than one hundred bioactive neuropeptides have been identified in vertebrates, varying in length from 3 amino acids, for thyrotropin-releasing hormone (TRH), up to several dozens of amino acids (82 for nesfatin-1). All neuropeptides are generated by cleavage of precursors of higher molecular weight that belong to three categories (Figure 1; Douglass et al., 1984). The first category includes mono-functional precursors that give rise to only one bioactive peptide flanked by one or two sequences called cryptic peptides, the function of which is generally unknown. Within these precursors, the bioactive peptide may be located at the N-terminal extremity upstream of the cryptic peptide as for neuropeptide Y (Cerdá-Reverter et al., 2000), in an intermediate position as for cholecystokinin (Beinfeld, 1997) or at the C-terminal extremity as for somatostatin and urotensin II (Vaudry et al., 2015; Figure 1A). The second category consists of mono-functional precursors with several copies of the bioactive peptide such as TRH, which exists in 5 copies within the same precursor in rat (Lee et al., 1988), resulting most likely from intragenic duplications during evolution (Figure 1B). The third category corresponds to multifunctional precursors giving rise to distinct bioactive peptides, the archetype being pro-opiomelanocortin (POMC), the precursor of adrenocorticotropic hormone (ACTH), melanotropic hormones (α-, β- and γ-MSH) and β-endorphin (Figure 1C; Nakanishi et al., 1979). The precursors of the first category can eventually be reclassified in the third category if an activity for one of the cryptic peptides is discovered (Figures 1A,C). In fact, sequences initially considered cryptic may sometimes prove to have a biological effect, as it was the case for nocistatin, present in the same precursor as nociceptin and having an inverse effect on pain transmission (Okuda-Ashitaka and Ito, 2000).

**FIGURE 2:**
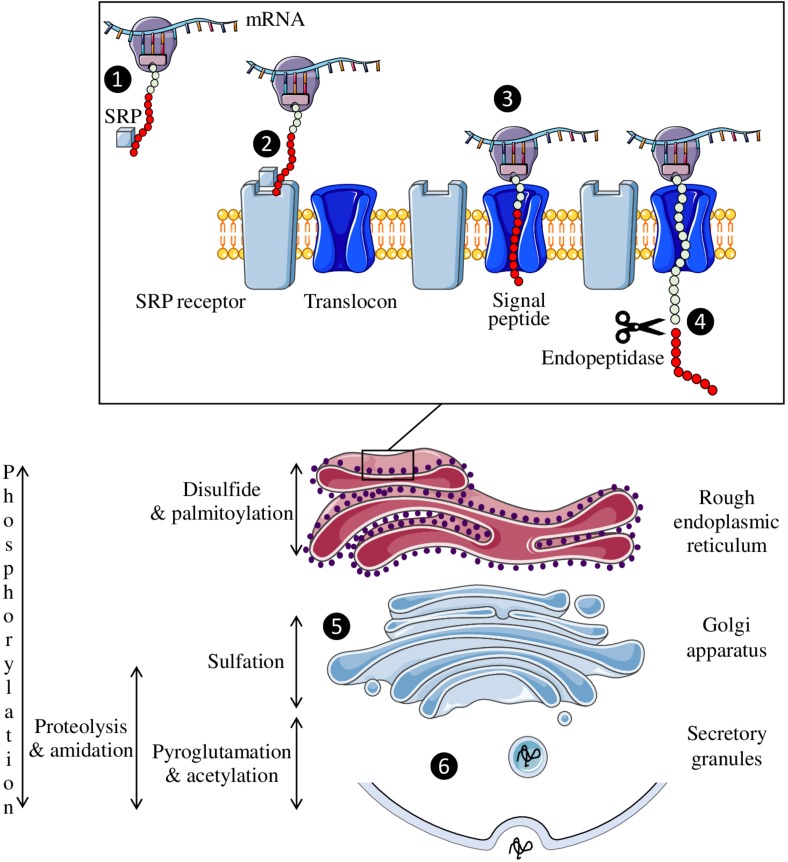
Neuropeptide biosynthesis. The signal recognition particle (SRP) is fixed to the complex formed by the peptide, the ribosome and the mRNA (❶) and then binds to its receptor on the endoplasmic reticulum membrane (❷). This allows the peptide to enter the reticulum through the translocon so that peptide synthesis can continue (❸). The signal peptide (red circles) is then cleaved off by an endopeptidase (❹). Once synthesized, the neuropeptide will pass through the Golgi apparatus to undergo post-translational modifications (❺) before being secreted in a regulated manner (❻).

The precursor polypeptide chains of neuropeptides exhibit several common structural features. First, a signal peptide consisting of a hydrophobic sequence of about twenty amino acids is located at the N-terminal extremity of the precursor. This signal peptide allows the translocation of the polypeptide into the lumen of the endoplasmic reticulum (Coleman et al., 1985). Once the signal peptide is translated by the ribosome, the complex binds to a ribonucleoprotein associated with an RNA molecule, the signal recognition particle (Figure 2). This particle then binds to its receptor located on the reticulum membrane, allowing the elongation and translocation of the preprohormone polypeptide into the reticulum to continue (Walter et al., 1984). During translation, the signal peptide is cleaved off by an endopeptidase, the signal peptidase.

Precursors translocated in the lumen of the reticulum and subsequently transported in cell compartments of the secretory pathway, undergo various post-translational modifications, such as the formation of a disulfide bridge between two cysteines, phosphorylation of a serine or threonine residue, tyrosine sulfation, octanoylation, C-terminal amidation, and N-terminal acetylation or pyroglutamylation. Each of these modifications can strongly affect the activity of the peptide and/or its resistance to enzymatic degradation. For example, sulfation of tyrosine in position 2 of cholecystokinin (Gigoux et al., 1998), N-octanoylation of the third serine residue of ghrelin (Kojima et al., 1999; Muccioli et al., 2001) or acetylation of the N-terminal serine of α-MSH (O’Donohue et al., 1981) significantly increases the affinity of these peptides for their receptors. Similarly, the presence of a disulfide bridge contributes to the spatial conformation of peptides, and its reduction usually leads to their inactivation as for oxytocin and urotensin II (Meraldi et al., 1977; Labarrère et al., 2003). These chemical modifications take place either during the transit of the precursor from the reticulum to the Golgi apparatus, or in the secretory vesicles. For example, disulfide bonds are formed in the endoplasmic reticulum, sulfation occurs in the Golgi apparatus and amidation, which concerns more than half of the neuropeptides, takes place in the secretory granules (Figure 2).

Once the precursors reach the trans-Golgi or secretory granules, they undergo specific cleavage by endoproteases called prohormone convertases (PCs) to give rise to biologically active peptides. There are 7 PCs in mammals: PC2 (Smeekens and Steiner, 1990), furin (van de Ven et al., 1990), PC1/3 (Seidah et al., 1991), PACE4 (Kiefer et al., 1991), PC4 (Nakayama et al., 1992), PC5/6 (Lusson et al., 1993) and PC7 (Tsuji et al., 1994). These 7 PCs share an affinity for substrates with basic amino acids (Fricker, 2012). The most common cleavage patterns consist of two basic amino acids such as a lysine-arginine and arginine-lysine doublets, two arginines or two lysines. PC1/3 and PC2, the most abundant enzymes, cleave the precursors downstream of these doublets and thus leave the two basic amino acids at the C-terminal extremity of the peptide. More rarely, the cleavage site may consist of a single basic amino acid as is the case for somatostatin-28 and the octadecaneuropeptide ODN (Barbaccia et al., 1990; Brakch et al., 1995). In the vast majority of cases, after cleavage, the basic residues are removed by carboxypeptidases (Fricker and Snyder, 1982; Song and Fricker, 1995), except for ODN and cortistatin (Ferrero et al., 1986; de Lecea et al., 1996). Two other PCs that do not cleave at basic sites have been characterized: subtilisin-kexin-isozyme (Seidah et al., 1999) and proprotein convertase subtilisin-kexin isozyme (Seidah et al., 2003). Differential expression of PCs depending on the cell type leads to tissue-specific cleavages of the precursors. An illustrative example of differential maturation is given by POMC that generates distinct bioactive peptides according to the tissues. Thus, in corticotrope cells of the adenohypophysis, where just PC1/3 is present, only half of the 8 potential cleavage sites are processed, giving rise to ACTH and β-LPH, whereas in neurons of the arcuate nucleus and melanotrope cells of the intermediate lobe of the pituitary, which express both PC1/3 and PC2, the maturation is complete and leads to the formation of α-MSH and β-endorphin (Bicknell, 2008). After cleavage and post-translational modifications, the bioactive peptide(s) is (are) stored in secretory vesicles which, upon depolarization of the cell, merge with the plasma membrane to release their content in the extracellular space.

Neuropeptides, therefore, represent a particular type of intercellular signaling molecules. Indeed, they are produced by nerve cells (and often by other cell types including endocrine cells, skin cells…), they derive from the specific cleavage of a preprohormone polypeptide harboring a signal peptide, and they are secreted in a controlled manner. Once released in the extracellular space, they act at low concentrations by binding to specific receptors before being degraded without reuptake, unlike neurotransmitters. These physical and biological characteristics can be exploited to identify novel neuropeptides. Since the first characterization of the neuropeptides oxytocin and vasopressin (Du Vigneaud, 1954), many research teams have set out strategies to discover novel neuropeptides. The techniques used for their identification are diverse and have evolved considerably over the last decades.

## Identification From a Biological Activity

**FIGURE 3:**
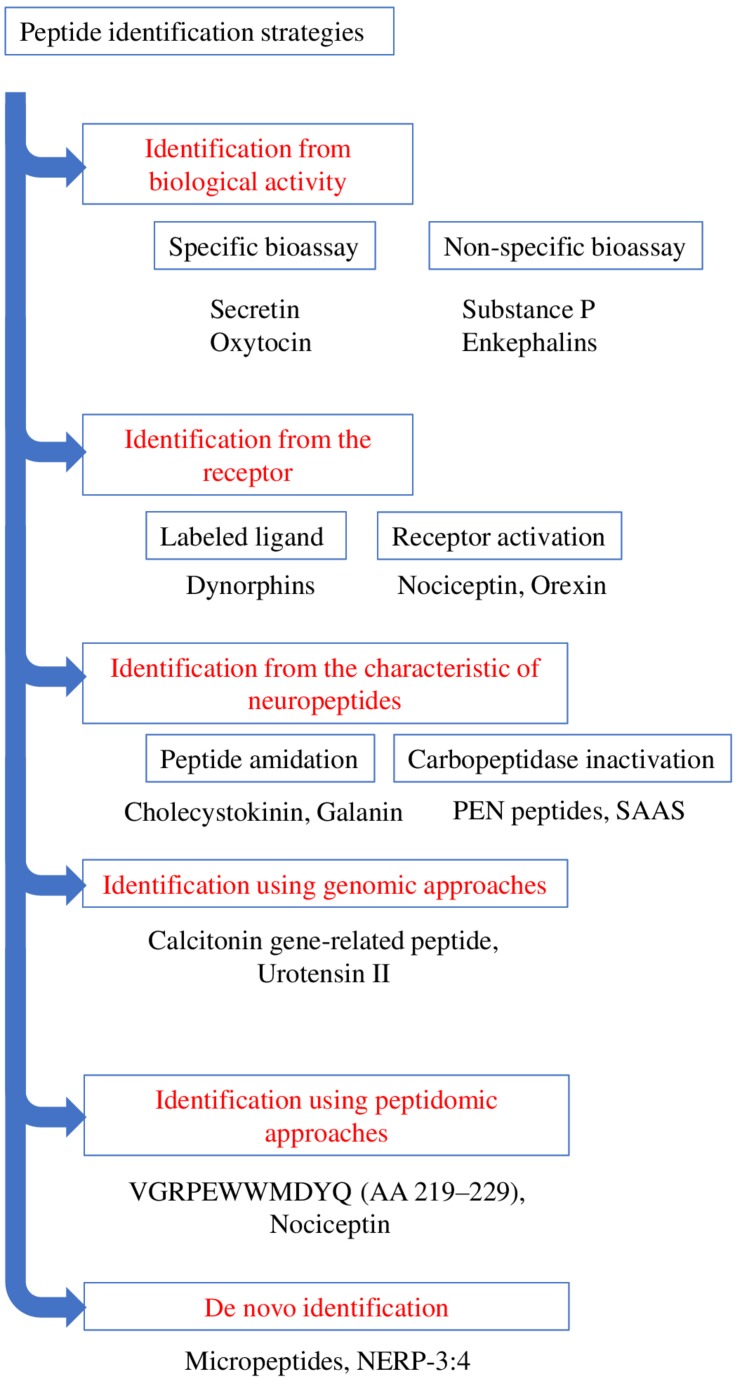
Schematic representation summarizing the various strategies of identification of bioactive peptides. Examples of the peptides identified through the various strategies are presented.

**FIGURE 4:**
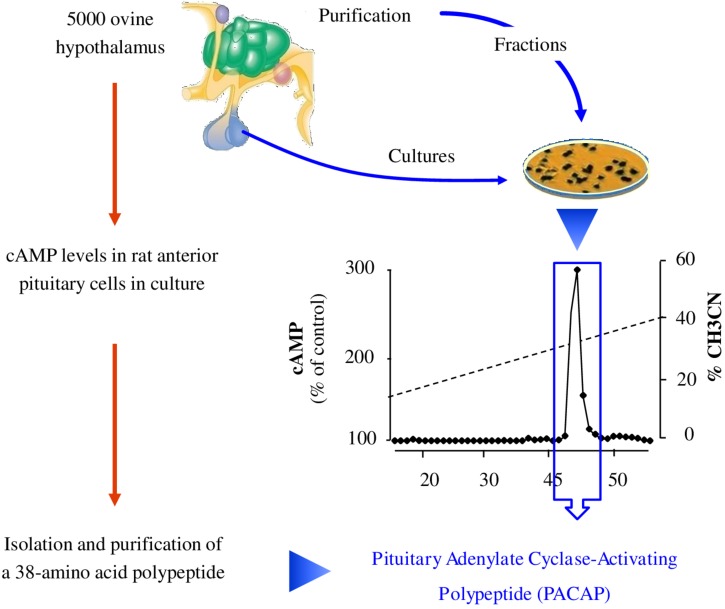
Strategy of identification of pituitary adenylate cyclase-activating polypeptide (PACAP). The team of Akira Arimura extracted peptides from 5000 ovine hypothalamic fragments and separated them according to their charge and hydrophobicity. A highly basic peptide different from any other known hypophysiotropic neurohormone was found to activate cAMP in cultured rat anterior pituitary cells. Characterization revealed a 38-amino acid peptide with 68% identity with VIP that they named pituitary adenylate cyclase-activating polypeptide or PACAP (Arimura, 2007). cAMP: cyclic adenosine monophosphate.

The initial method employed to discover bioactive peptides relies on a biological test, which consists of measuring the effect of tissue extracts, most often brain extracts for neuropeptides, on a physiological parameter. These extracts are then purified until a single compound is isolated. Two types of approaches can be distinguished for bioassays depending on whether the activity being tested corresponds to the “main function” of the peptide or to a mere pharmacological effect (Figure 3). Regarding the main function, it is through this approach that the first neuropeptide, oxytocin, was isolated by Du Vigneaud et al. (1953). Hypothalamic hypophysiotropic neuropeptides such as TRH, gonadotropin-releasing hormone and somatostatin have been discovered via the same approach by studying the ability of hypothalamic extracts to modulate the release of thyrotropin (Burgus et al., 1969), luteinizing hormone, follicle-stimulating hormone (Schally et al., 1971) and growth hormone (Brazeau et al., 1973). A similar strategy was also used to identify pituitary adenylate cyclase-activating polypeptide (PACAP) from a sheep hypothalamic extract by measuring the activation of adenylate cyclase in pituitary cells, hence the name of this peptide (Miyata et al., 1989; Figure 4).

Besides, pharmacological (non-specific) activity tests were used to isolate other peptides such as substance P or enkephalins. For instance, the purification of substance P was carried out by measuring the effects of gut extracts, and later of brain extracts, on the contraction of intestine smooth muscles (Euler and Gaddum, 1931). However, it took four decades before the sequence of the peptide was determined (Chang et al., 1971). Regarding enkephalins, the test consisted in monitoring the effect of brain extracts on electrically evoked contractions of the mouse vas deferens and guinea pig ileum (Hughes et al., 1975). These inhibitory effects were completely antagonized by the opioid receptor antagonist naloxone.

Even though these approaches by biological tests have led to the identification of several neuropeptides, they are not devoid of drawbacks. In particular, the successive steps of purification and the activity tests until reaching a single bioactive compound can be very long. Furthermore, once a peptide such as PACAP has been characterized, decades of research are still necessary to identify its numerous functions (Vaudry et al., 2000, 2009) and decipher its mechanisms of action (Vaudry et al., 1998, 2002).

## Identification From the Receptor

Large-scale cloning of G-protein coupled receptors (GPCRs), has led to the identification of numerous so-called orphan receptors, that is, receptors whose endogenous ligands have not yet been identified. The human genome encompasses ∼800 genes encoding GPCRs of which 448 are sensory receptors (Mombaerts, 2004). Among the remaining ∼350 GPCRs, ∼140 still have no identified ligand (Stockert and Devi, 2015). Since about 50% of non-orphan GPCRs are activated by (neuro)peptides, a quick extrapolation indicates that approximately 70 orphan GPCRs should recognize one or several biologically active peptides(s) as natural ligand(s) (Alexander et al., 2011). These peptide-binding GPCRs thus represent very attractive targets for the identification of novel neuropeptides through a reversed pharmacology approach *via* the screening of tissue extracts or synthetic peptide libraries. The search for the endogenous ligand(s) of an orphan GPCR necessitates the stable or transient expression of the receptor cDNA in a cell line in order to screen tissue extracts. The pairing of a potential neuropeptide to a receptor of interest can be carried out through two types of approaches (Figure 3). The first one uses a labeled ligand (most often radioactive) whose binding to the receptor is displaced by the endogenous ligand (Figure 3). In this case, an already known neuropeptide is used to identify novel related endogenous peptides that bind to the same receptor. This approach allowed the identification of several neuropeptides from prodynorphin, including different types of dynorphins (Pert et al., 1977; Goldstein et al., 1979). The main drawback of this technique is the requirement to label a peptidic ligand by adding a radioactive iodine atom to a tyrosine without dramatically altering its affinity. Alternatively, it is possible to label peptides with tritiated or deuterated atoms which do not impair the structure of the molecules (Allen et al., 1982). The second method focuses on the receptor activation, studied by measuring a physiological parameter such as cytosolic calcium level, second messenger formation, acidification, … (Figure 3). It is through this approach that nociceptin and orexin were identified (Meunier et al., 1995; Reinscheid et al., 1995; Sakurai et al., 1998). Variants of this approach include imaging techniques that monitor *e.g.*, the internalization of the fluorochrome-labeled tagged receptor (De Mota et al., 2004) or microcalorimetry that reflect the interaction between the receptor and its ligand through quantification of conformational change-induced temperature variations (Jerabek-Willemsen et al., 2014).

## Identification From Biochemical Characteristics of Neuropeptides

The methods presented above focus on the discovery of neuropeptides *via* their activity, either at the tissue or cell level. Besides this functional-based strategy, it is possible to identify novel neuropeptides according to their biochemical features. The main characteristic signature is peptide amidation, which can be studied by chemical precipitation steps of the amidated fragments prior to their purification. This approach has been used successfully by Viktor Mutt’s team to isolate various neuropeptides, some of which had already been identified in other species, such as cholecystokinin (Tatemoto et al., 1984), while others were completely novel such as neuropeptide Y (Tatemoto et al., 1982) and galanin (Tatemoto et al., 1983). The main difficulty with this approach comes from the very large amount of material required: it took for example 400 kg of pig brain to purify and characterize neuropeptide Y. It should be noted that the continuous improvement of analytical instrument sensitivity can now greatly facilitate the identification steps. However, only about half of the known neuropeptides are amidated (Kim and Seong, 2001), implying that the other half cannot be identified by this technique. Other biochemical characteristics can be used to identify neuropeptides within precursors, such as the presence of cleavage sites for PCs, which has allowed to discover new peptides from precursors already known as it was the case for nocistatin (Okuda-Ashitaka et al., 1998). Finally, a method more rarely used is based on the activity of the enzymes involved in peptide biosynthesis, such as carboxypeptidase E (Hook and Loh, 1984). After cleavage of the precursor by PCs, carboxypeptidase E acts to remove the basic amino acids located at the C-terminal position. Inactivation of carboxypeptidase E results in the accumulation of an almost mature form of the putative neuropeptide that can then be more easily purified and sequenced. Such a strategy led to the identification of PEN peptides, derived from a precursor close to the granins, SAAS (Fricker et al., 2000), whose receptor has not yet been identified. This technique has the same limitations as the previous ones, namely not all neuropeptides are substrates of carboxypeptidase E. Moreover, it should be kept in mind that all these approaches to identify neuropeptides are only the first steps of a long research process since once the candidate has been purified and sequenced, it is necessary to identify its receptor(s) in order to investigate into details its functions.

## Identification Using Genomic Approaches

Comparison of cDNA or genomic sequences between distant species has allowed the discovery, in mammals, of several biologically active peptides previously identified in non-mammalian species. For instance, the cDNA of the calcium-regulating hormone stanniocalcin has been initially cloned in teleost fish (Butkus et al., 1987) and the human ortholog has been identified only a decade later (Olsen et al., 1996; Chang et al., 1998). Similarly, the cDNA of the hypertensive peptide urotensin II (UII) has been first characterized in frog, and has been subsequently used to identify the human UII cDNA sequence (Coulouarn et al., 1998). Theoretically, invertebrate genomic sequences could also be exploited for the identification of vertebrate neuropeptide cDNAs (Elphick et al., 2018). The comparative approach has also been used successfully at the peptidic level. Thus, melanin-concentrating hormone (MCH) which has been initially identified in fish as a pituitary hormone (Kawauchi et al., 1983) was subsequently sequenced in a rat hypothalamic extract (Vaughan et al., 1989) before its cDNA was finally cloned in rat (Nahon et al., 1989) and human (Presse et al., 1990).

The detection of possible alternative splicing events may also lead to the discovery of novel regulatory peptides. This was the case with the calcitonin primary transcript that, through tissue-specific processing, can give rise to two distinct mRNAs *i.e.*, calcitonin mRNA in thyroidal C cells and calcitonin gene-related peptide (CGRP) mRNA in neuronal cells (Amara et al., 1982; Rosenfeld et al., 1983). CGRP has thus been originally identified as an alternative splicing product of the calcitonin gene.

Genome sequencing of representative species of the different phyla has allowed the development of libraries containing almost all the genomic sequences of these species. However, the lack of gene annotations has made it necessary to create powerful computer tools to screen these libraries for the identification of novel neuropeptides. The developed softwares employ various strategies. First, sequence alignments can be used to compare non-annotated genes in one species with genes encoding neuropeptides in another species (Siepel et al., 2005). Thus, the genes that are conserved during evolution can be identified and are likely to exert an important function (Sonmez et al., 2009). Then, screening of cDNA libraries can identify sequences having common patterns with known neuropeptide families, such as the RFamide motif (Nathoo et al., 2001). Finally, it is possible to rely on the characteristics of neuropeptide precursors to screen genomic libraries for candidate sequences (Hewes and Taghert, 2001). These approaches use mathematical models like the Markov model, assigning a score to the sequences according to their correspondence with each of the criteria. It is then necessary to verify the expression of these sequences in neuronal cells and to look for an activity of the putative peptide. Nevertheless, *in silico* analysis can lead to a large number of predicted peptide candidates, which may require the synthesis of an important peptide library for subsequent biological screening (Liu et al., 2008; Shemesh et al., 2008; Kliger, 2010). But some methods can help to reduce this number of candidates by using gene expression mapping to create peptide-receptor pairs (Williams et al., 2017).

It should be noted that these genomic techniques of identification of neuropeptides are progressively being supplanted by new and more efficient approaches. In particular, the evolution of mass spectrometry techniques has allowed the development of global peptidomic approaches for the discovery of novel biologically active peptides.

## Identification Using Peptidomic Approaches

**FIGURE 5:**
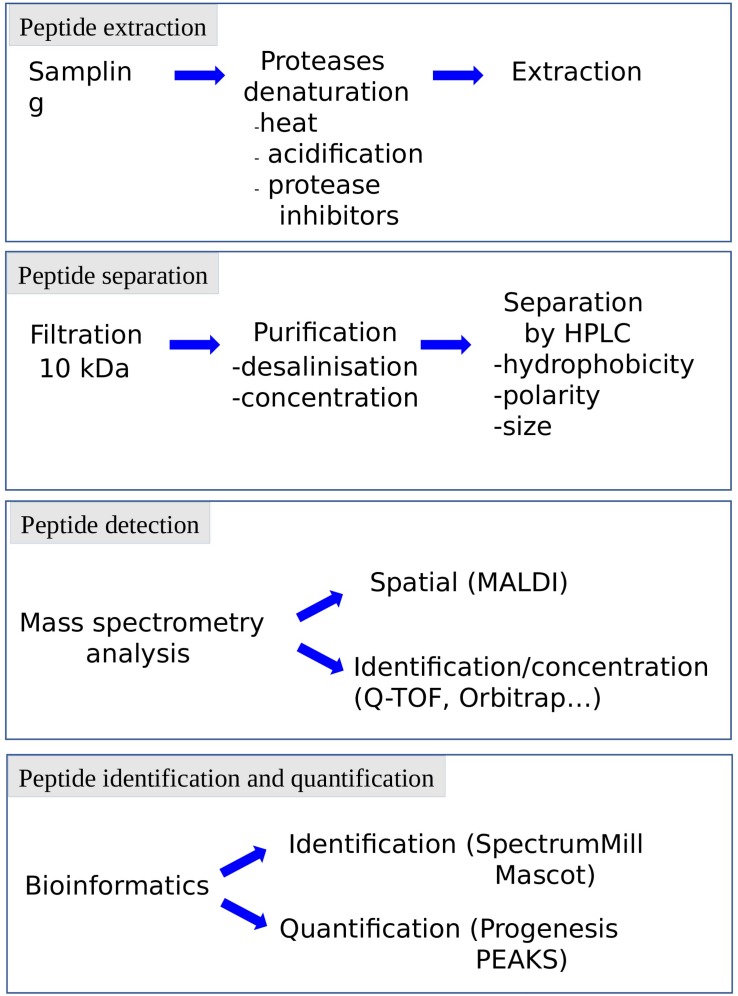
Representation of the various steps of peptidomic studies. Each step can be adapted to the specificity of the peptide, the complexity of the matrix and the information needed (e.g., identification, quantification). HPLC: high pressure liquid chromatography.

The term peptidomic appeared for the first time in 2001 in a study using mass spectrometry to develop a peptide profile in locusts (Clynen et al., 2001). Peptidomic methods (Figure 3) require a smaller amount of starting material and a shorter analysis time by reducing the successive purification steps. In addition, they make it possible to obtain more information on the candidate peptides, such as their sequence or the presence of post-translational modifications. Peptidomic studies are conventionally carried out in 4 steps, namely (i) peptides extraction, (ii) separation, (iii) detection and (iiii) identification and quantification (Figure 5).

The preservation of the peptides in their native state is crucial for peptidomic studies and must begin as soon as the sample is collected, to avoid the breakdown of proteins into peptides (false positives) or the degradation of the bioactive peptides (false negatives). Protection of the samples can be achieved by heat denaturation of the proteolytic enzymes, acidification and/or addition of protease inhibitors. This can be done manually or using specific equipment such as the heat stabilization system Denator^®^. The extraction step has been refined and becomes more accessible and reproducible by the outbreak of commercial extraction tools. This step may include the use of centrifugal filters to remove larger proteins. The cut-off threshold conventionally used in peptidomics is 10 kDa, to get rid of proteins with a size greater than one hundred amino acids. The purification procedure is complemented using chromatography devices such as ZipTips^®^ or Sep-Pak^®^ to desalt, concentrate and purify samples.

The separation step, carried out upstream of the mass spectrometer, has been improved to allow the analysis of complex mixtures. Indeed, while it is possible to identify peptides via direct analysis of the sample by mass spectrometry, the presence of peptides and proteins of different molecular weight and variable concentrations in a complex matrix necessitates to include one or more separation steps upstream of the analyzer. This limitation can be circumvented by coupling the mass spectrometer to a separation method allowing the successive analysis of the various compounds of the sample by mass spectrometry. High performance liquid chromatography (HPLC) makes it possible to separate the peptides according to their hydrophobicity, polarity and/or size. The miniaturization of the columns (nanoHPLC) and the increase of the pressure (ultraHPLC), permit to increase the system sensitivity and resolution, and thus to improve the analyte separation. These ameliorations contribute to significantly reduce the amount of samples needed.

Peptide identification and quantification by mass spectrometry has also been considerably improved in recent years, particularly with the implementation in the proteomic platforms of devices such as high-resolution accurate-mass *Orbitrap* mass spectrometers which surpass the MALDI-TOF MS systems. The sensitivity level, acquisition rate, and range of analyzable masses have steadily increased through progress of ionization, ion trapping, and fragmentation (Eliuk and Makarov, 2015). In particular, the expansion of peptidomic techniques allowing the accumulation of information on peptide sequences and post-translational modifications has been facilitated by combination of fragmentation modes such as CID (Collision Induced Dissociation), HCD (Higher-Energy Collision Induced Dissociation) and ETD (Electron Transfer Dissociation) (Hayakawa et al., 2013). Peptide identification and quantification has also been made easier with advances in computer science. Indeed, the increase in the amount of data generated, that can reach for each experiment several tens of gigabytes, implies the development of bioinformatic databases and algorithms. In particular, data analysis is now facilitated by the development of powerful softwares to perform label-free quantitation (such as Progenesis), or database queries (such as Spectrum Mill or Mascot; Martens et al., 2005).

**FIGURE 6:**
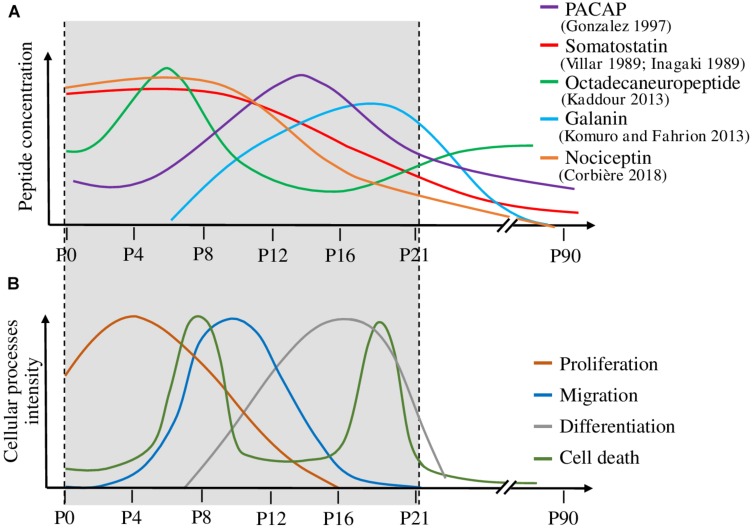
Correlation between the expression level of various peptides and the mechanisms occurring during cerebellar cortex development. **(A)** Variation of five peptide concentrations during cerebellar development. **(B)** Temporal evolution of the cellular processes involved in cerebellar development, i.e., proliferation, migration, differentiation and cell death. Peptide concentrations are high during the developmental processes on which they act. P: postnatal day. PACAP: pituitary adenylate cyclase-activating polypeptide.

**FIGURE 7:**
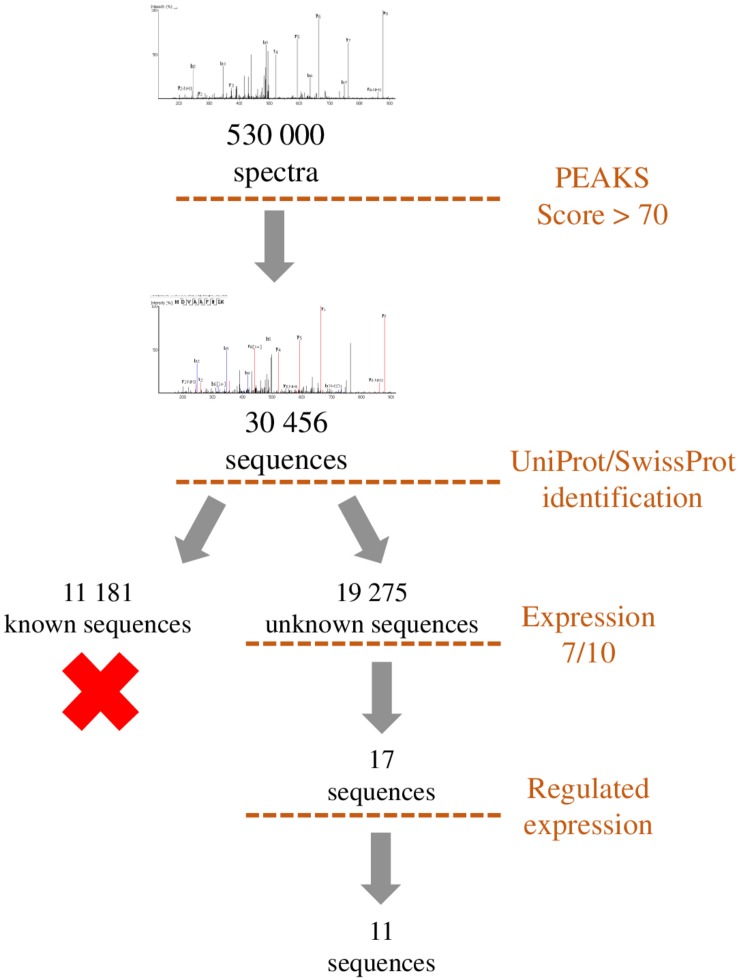
Representation of the workflow used to filter the peptides identified by *de novo* sequencing. Sequences with a PEAKS score higher than 70 were compared to UniProt/SwissProt database in order to exclude peptides originating from already known precursors. Remaining sequences were filtered by occurrence among samples (>7/10) and regulation during development (>2). This workflow led to the selection of 11 sequences which correspond to putative peptides.

If peptidomic techniques were initially set up and still contribute to identify novel neuropeptides *de novo*, quantitative peptide profiling by mass spectrometry (also called differential peptidomics) is now used to screen and quantify already characterized peptides in tissues where they were not known to be present and in different conditions. Such an approach was used to characterize peptides with daily regulation in the rat suprachiasmatic nucleus and led to the discovery that the peptide VGRPEWWMDYQ (AA 219–229), derived from proenkephalin A, is significantly increased at night-time (Lee et al., 2013). Still in the brain but during development, peptides acting on the ontogenesis of the cerebellar cortex (the external part of the cerebellum) often exhibit a specific pattern of expression with, in rodents, high expression over the first 2 postnatal weeks, and a decline at adulthood (Figure 6; Inagaki et al., 1989; Villar et al., 1989; Malagon et al., 1993; Tatsuno et al., 1994; Kaddour et al., 2013; Komuro and Fahrion, 2013). Based on this observation, it was possible to identify additional peptides exhibiting such a bell-shape expression profile (Corbière et al., 2018). Using a neuropeptide database, 33 peptides were identified in the cerebellum by mass spectrometry, among which 4 had a high expression level during development, which then decreased at adulthood. Further studies conducted on one of them, *i.e.*, nociceptin, confirmed that not only the peptide but also the expression of its precursor gene and of its receptor are regulated during development. As the developing cerebellar cortex is composed of a dozen of cell types spread in 4 different well defined layers, an attempt was conducted to identify nociceptin by a MALDI-imaging approach. Although 8 compounds differentially expressed during cerebellar development could be detected through this approach, no peptide with an m/z corresponding to nociceptin could be unequivocally identified. Two peptides whose expression increased with cerebellar maturation may correspond to cerebellin-1 and [des-Ser1]-cerebellin but the measurement accuracy and the lack of fragmentation data show the limits of this approach (Belov et al., 2017). In fact, even if there are many developments around MALDI imaging, to date, only few bioactive peptides have been clearly identified through this approach (Ljungdahl et al., 2011; Hanrieder et al., 2012; Sui et al., 2017; Huber et al., 2018). To circumvent the problem, laser microdissection of the tissue coupled with real time PCR analysis was used to determine that both nociceptin and its receptor genes were mostly expressed in the internal granular layer of the cerebellar cortex, which mainly contains granule neurons. Subsequent functional studies showed that nociceptin exerts a neurotrophic effect on those granule neurons by increasing their survival and promoting their differentiation (Corbière et al., 2018; Figure 7). In an attempt to obtain information regarding cell specific peptide content, single cell neuropeptide profiling is a new challenge which has started to be developed (Jiménez et al., 2006; Neupert et al., 2012; Do et al., 2018).

## *De novo* Identification

To identify unknown peptides, *de novo* peptide sequencing of experimental data obtained by mass spectrometry can be carried out using softwares such as PEAKS. Nevertheless, some filters have to be applied to reduce the number of obtained sequences. A first step can be to eliminate all the sequences already present in SwissProt and/or UniProt databases coding for already known proteins and then to only retain sequences expressed recurrently and potentially regulated by a treatment, a disease or a developmental process. This allows the selection of several candidate peptides that are potentially bioactive in order to reverse-transcribe their sequence to identify the gene from which they are derived. Nevertheless, it remains to be determined whether some of these sequences can correspond to bioactive peptides. This can be achieved by looking at some selection criteria such as the presence of a sequence coding for a signal peptide upstream of the peptide of interest, the existence of basic doublets at the N- and C-terminal ends which may correspond to cleavage sites by PCs, and evolutionary conservation. If the peptide is under certain conditions regulated, it can be looked at the regulation of its gene expression and, finally, possible functions should be investigated. This complex approach, which requires support from bioinformatics teams, has led to the identification of Neuroendocrine regulatory peptides (NERP-3; Sasaki et al., 2009) and other peptides derived from the VGF sequence (Sasaki et al., 2010). New methods of identification are also used to reduce the search space for spectral matching to improve the discrimination between correct peptide identification and false hit, and thus decrease the number of sequences (Hayakawa et al., 2013). Proteogenomics approaches are now also emerging to identify functional micropeptides produced from sORFs within cells of diverse species (Yeasmin et al., 2018). This approach often leads to a large number of peptide candidates that can nowadays be synthesized with dedicated robotic systems such as the Apex 396HT^®^ Peptide Library Synthesizer.

The strategies developed in order to identify novel bioactive peptides have evolved considerably during the past decades. The successful but laborious pioneer tissue purifications combined to biological tests or immunoassays, that were rewarded by the attribution of several Nobel Prizes (Du Vigneaud, 1954; Guillemin, 1977; Schally, 1977; Yalow, 1977), are now supplanted by reverse pharmacology and peptidomic approaches which have already led to the discovery of several bioactive peptides. Owing to the raising interest of the pharmaceutical industry for peptide-based drugs (Fosgerau and Hoffmann, 2015; Henninot et al., 2018), the search of novel biologically active peptides is currently a very active domain. Rapid developments in bioinformatics, combined with powerful analytical methods and highly sophisticated mass spectrometers, should allow to de-orphanize the dozens of still orphan peptide-targeted GPCRs in vertebrates. These novel bioactive peptides will undoubtedly contribute to the rational design of innovative compounds either peptidergic analogs or small molecule-base drugs.

## Author Contributions

AC wrote the first draft of the manuscript. DV and HV corrected and completed the manuscript. PC, M-LW-B, TL, AL, and CP gave their technical knowledge to correct parts of the manuscript. All authors contributed to the manuscript revision, read and approved the submitted version.

## Conflict of Interest Statement

The authors declare that the research was conducted in the absence of any commercial or financial relationships that could be construed as a potential conflict of interest.
